# Patients' and Therapists' Views of Integrated Online CBT for Depression

**DOI:** 10.1111/hex.70002

**Published:** 2024-08-21

**Authors:** Fiona Fox, Nicola Wiles, David Kessler, Debbie Tallon, Laura Thomas, Christopher Williams, Roz Shafran, Paul Lanham, Katrina Turner

**Affiliations:** ^1^ NIHR ARC West Bristol UK; ^2^ Population Health Sciences, Bristol Medical School University of Bristol Bristol UK; ^3^ Centre for Academic Mental Health, Population Health Sciences, Bristol Medical School University of Bristol Bristol UK; ^4^ Institute of Health and Wellbeing University of Glasgow Glasgow UK; ^5^ Five Areas Ltd Titan Enterprise Business Centre Clydebank, West Dunbartonshire UK; ^6^ Great Ormond Street Institute of Child Health University College London London UK; ^7^ Public and Patient Involvement Representative London UK

**Keywords:** CBT, cognitive behavioural therapy, depression, online therapy, qualitative, typed therapy

## Abstract

**Background:**

CBT is an effective treatment for depression, but access varies across the United Kingdom. Online CBT increases access. The INTERACT platform was designed to support patient engagement in CBT, enabling therapists to deliver high‐intensity CBT via typed instant messaging and allowing patients and therapists access to an integrated online library of resources during and between sessions.

**Methods:**

The INTERACT trial aimed to evaluate this integrated approach to delivering CBT for primary care patients with depression. A nested qualitative study was conducted within the trial. Interviews were conducted with 20 patients who received the intervention, 9 therapists who delivered it and 3 therapist supervisors. Data were analysed using thematic analysis.

**Results:**

The combination of receiving support from a therapist and having access to integrated online CBT resources enabled patients to better manage their depression. Platform benefits included the opportunity to review transcripts to clarify how to complete homework tasks and track progress in managing their depression. The typing process allowed reflection and a focused discussion. However, less could be covered than during an in‐person session, which reduced therapists’ expectations around goal setting. Patients who did not complete therapy struggled with the typing and found the CBT approach too demanding.

**Conclusion:**

Findings highlight the importance of establishing patient and therapist goals and expectations about what can be achieved in CBT mediated by typing. Some patients are comfortable communicating via typing and are motivated to utilise online resources in between sessions. Exploring the benefits and challenges of typed CBT with patients will enable them to make an informed choice about referral for this novel approach to therapy.

**Patient or Public Contribution:**

Patients, service users and members of the public were involved in the study design and management. Substantial pilot work gathered stakeholder feedback and informed the design of the intervention, before undertaking the RCT. Coauthor P.L. is a service user representative co‐applicant and member of the management group responsible for developing the intervention and the trial. Two PPI members sit on the Independent Steering Committee. PPI members provided valuable feedback on the study resources and documents.

## 1 Introduction

Cognitive behavioural therapy (CBT) is an effective treatment for depression [1, 2], but provision of, and access to, CBT via the NHS in England is variable [3]. Online CBT has improved accessibility and availability. Delivery models vary in the degree of therapist involvement, from fully self‐guided programmes with access to CBT resources or CBT‐based life skills resources with or without automated reminders/prompts [4, 5] to therapist‐supported sessions mediated by real‐time instant messaging, phone or video support but which do not offer access to resources such as worksheets [6, 7]. Whilst online CBT can be provided at lower cost than in‐person therapy [5], challenges include poor uptake and adherence, potentially crucial aspects of CBT being omitted (e.g., identification of conditional beliefs and detailed formulations) [8]. These factors might explain its relatively modest and short‐term effects [8, 9]. Programmes where therapists deliver CBT sessions via real‐time instant messaging can be as effective as in‐person CBT [10, 11], and trials evaluating a blended model of online self‐guided CBT with some therapist input have shown it to be effective in treating major depressive disorder [7].

Access to CBT resources (such as worksheets), both during and between sessions is crucial to support homework tasks [12] where people apply or test out new skills, the completion of which is an important mediator of treatment outcome [13, 14]. However, only some existing online CBT platforms include accessible CBT resources. Given that over 88% of UK adults have a smartphone [15], there is a growing opportunity to provide online CBT resources via mobile devices to increase accessibility and facilitate more immediate and discrete recording of thoughts and experiences [16].

The INTERACT platform was designed to support patient engagement in CBT via a desktop, laptop, tablet or smartphone. The aim was to provide integrated CBT, which involved an online therapy platform within which accredited CBT therapists could deliver high‐intensity CBT via typed instant messaging. Both patients and therapists could access an online library of resources (worksheets, videos and information sheets) during and between sessions [17]. The INTERACT platform was developed iteratively via a process of design [18] and pilot evaluation with input from CBT therapists and patients with depression [18, 19].

INTERACT, a randomised controlled trial (RCT), was conducted to evaluate the clinical and cost‐effectiveness of integrated online CBT for primary care patients with depression. The trial aimed to evaluate whether those receiving the intervention had reduced depressive symptoms and improved quality of life over 12 months, compared with patients who received usual GP care. The trial included a nested qualitative interview study with therapists, their supervisors and patients to explore their views and experiences of integrated online CBT. This paper reports findings from these interviews to provide insight into the acceptability of the intervention. The findings on clinical and cost‐effectiveness will be reported separately.

## 2 Materials and Methods

The INTERACT trial was based in three trial centres: the University of Bristol (coordinating centre), University College London and the Universities of Hull/York [17]. Patients with depression were recruited from primary care (67 general practices). PPI members provided valuable feedback on the recruitment materials (patient information sheet, invitation letter and consent form). Participants were eligible to take part if they were aged 18 years or over, scored 14 or more on the Beck Depression Inventory (BDI‐II) [20] and met ICD‐10 criteria for a primary diagnosis of depression [21] at the baseline assessment with the researcher. Those eligible were randomised to either (1) integrated online CBT or (2) usual GP care.

Therapists employed on the trial were all CBT therapists with British Association for Behavioural and Cognitive Psychotherapies (BABCP) accreditation. They received training in using the platform and, whilst delivering the intervention, received weekly supervision from an accredited CBT therapist.

Participants randomised to the intervention group received nine high‐intensity CBT therapist‐led sessions via the online platform, with (up to) a further three sessions if therapists deemed clinically appropriate. The first session lasted up to 90 min and took place face to face via videocall (independent of the INTERACT platform) with the aim of building an initial relationship with the patient. Subsequent 50‐min sessions took place on the platform, via typed instant messaging. Therapists were encouraged to use only instant messaging after the first session but had the option of using phone or videocall if there was a specific reason, such as concern around patient risk. The library of CBT resources (worksheets, information sheets and videos) supported the standard Beck depression protocol [20]. The platform resources could be used by patients during and between online sessions and until their 12‐month trial follow‐up. During sessions, worksheets could only be filled in by patients, but therapists were able to advise on worksheet completion via the instant messaging chat. Patients' between‐session activities were automatically logged on the platform. In between sessions, worksheets could be shared with therapists, which they could comment on. Agendas, transcripts and session summaries could be reviewed by both parties. During supervision, therapists and their supervisors could review session transcripts, worksheets and client depression scores (PHQ‐9).

### 2.1 Recruitment to the Qualitative Study

To achieve the most complete picture regarding the acceptability of integrated online CBT to those receiving this treatment and involved in its implementation in practice, we interviewed patients, therapists and supervisors.

#### 2.1.1 Patient Recruitment

Trial participants (*n* = 451) provided consent to be contacted about the qualitative interviews as part of their baseline consent form. The qualitative researcher F.F purposefully sampled participants who had been randomised to receive integrated CBT, once they had completed their 6‐month trial (primary) outcome measures. Patients were sampled from the three trial sites and across the trial therapists to achieve maximum variation in relation to age, gender, depression severity, treatment response, adherence to treatment and therapeutic alliance [22]. F.F. contacted individuals sampled for interview, outlining the purpose of the interview and providing the participant information sheet and consent form specific to the qualitative study by email. A time for the telephone interview was agreed with those who were willing to participate.

#### 2.1.2 Therapist and Supervisor Recruitment

When joining the trial, all therapists (*n* = 9) and clinical supervisors (*n* = 3) gave written consent to take part in an interview. Therapists were interviewed when they had delivered the intervention to most of their trial patients. Supervisors were also interviewed at this point, as they had completed most of their supervision. See Table 1 for therapist and supervisor characteristics.

**Table 1:** Therapist and supervisor sample characteristics.

Characteristic		
Gender (female), *n* (%)	11	(91.7)
Age at consent (years), mean (SD)	43.8	(8.9)
Professional background, *n* (%)		
CBT therapist^a^	10	(83.3)
Clinical psychologist	2	(16.7)
Years practising as CBT therapist, median [IQR]	11	[4.5, 12]
Experience of supervising other CBT therapists, *n* (%)		
Yes	6	(50)
Technology previously used to deliver/support CBT^b^, *n* (%)		
None	4	(33.3)
IESO	5	(41.7)
Beating the blues	4	(33.3)
Other	4	(33.3)

^a^ Three of the CBT therapists also had a nursing background.

^b^ Could select more than one option.

#### 2.1.3 Interviews

Semi‐structured interviews with patients were conducted by F.F. via telephone. Interviews with therapists and supervisors were conducted by F.F. and K.T. via telephone or videocall (MS Teams). Verbal consent was agreed immediately before the interview. For each sample (patients, therapists, supervisors), a separate topic guide was used. Guides were developed according to the study aims, relevant literature and team discussions and revised as interviews progressed. They included similar questions to facilitate the triangulation of patients', therapists' and supervisors' accounts. All the interviews were audio recorded, transcribed verbatim and anonymised by F.F.

#### 2.1.4 Analysis

The data were initially analysed separately in two sets: (1) patients and (2) therapists and supervisors (grouped together due to their experience of therapy delivery). Analysis of all data followed the same thematic process [23]. Transcripts were read by F.F. to identify themes and develop a coding frame. K.T. read a subset of transcripts, applied this coding frame and then met with F.F. to review and amend it. The coding frame reflected themes identified inductively from reading the transcripts and areas of questioning in the topic guides (see Supporting Information). Once the coding frame had been agreed, F.F. imported the transcripts into the software package NVivo to allow electronic coding and data retrieval. Researchers F.F. and K.T. monitored and reviewed the data to ensure they yielded adequate ‘information power’ [24]. This was determined by the quality of interview dialogue with the study participants and the extent to which it enabled the team to answer the research questions.

## 3 Findings (results)

A total of 20 patients were interviewed, aged between 21 and 69 years (mean age: 43.6 years). Ten were male and 10 were female, and they had completed between 2 and 12 therapy sessions (median number of sessions was 9 [IQR: 4.5, 9.5]). Twelve patients had completed therapy (defined as having at least nine sessions of therapy or reaching an agreed end), and eight patients had not completed therapy (Table 2). Patient interviews were conducted between December 2021 and May 2023. Mean duration was 44 min (range: 27–58 min).

**Table 2:** Patient sample characteristics.

Characteristic		
Gender (female), *n* (%)	10	(50)
Age at baseline (years), mean (SD)	43.6	(17.2)
Centre, *n* (%)		
A	11	(55)
B	5	(25)
C	4	(20)
Baseline BDI‐II score, mean (SD)	29	(10.5)
Baseline ICD‐10 primary diagnosis of depression, *n* (%)		
Mild	2	(10)
Moderate	11	(55)
Severe	7	(35)
Baseline ICD‐10 secondary diagnosis, *n* (%)		
Mixed anxiety and depressive disorder (mild)	3	(15)
Mixed anxiety and depressive disorder	3	(15)
Specific (isolated) phobia	2	(10)
Agoraphobia	1	(5)
Generalised anxiety disorder	8	(40)
Panic disorder	3	(15)
Number of therapy sessions attended, mean (SD)	7.5	(3.2)
CBT completers/non‐completers, *n* (%)		
Completers	12	(60)
Non‐completers	8	(40)
Duration of current episode of depression at baseline, *n* (%)		
Between 2 weeks and 6 months	1	(5)
Between 6 months and 1 year	3	(15)
Between 1 and 2 years	5	(25)
Between 2 years and 5 years	1	(5)
More than 5 years	10	(50)
Previous history of depression, *n* (%)		
Yes	18	(90)
Employment status, *n* (%)		
Employed full‐time	11	(55)
Employed part‐time	4	(20)
Student	2	(10)
Retired	1	(5)
Unemployed due to ill‐health	2	(10)
Highest educational qualification, *n* (%)		
Degree or equivalent	6	(30)
HNC, HND, SVQ (Levels 4 or 5) or RSA higher diploma	4	(20)
A‐level, higher grade, or equivalent, or RSA advanced diploma	4	(20)
GCSE, standard grade, O‐level or equivalent, or RSA diploma	6	(30)

Nine therapists and three supervisors were interviewed. The therapists had delivered integrated online CBT to between 10 and 35 trial patients (mean number of patients per therapist = 23). Eight therapists were female and one was male (mean age = 43.8 years). Before the trial, five therapists and one supervisor had previously delivered CBT via typed instant messaging. Interviews with therapists and supervisors were conducted between October 2021 and May 2023. Therapist interviews lasted an average of 60 min (range: 39–75 min), and supervisor interviews lasted an average of 33 min (range: 27–40 min).

Below, findings from both patients and therapists are presented together, reflecting key themes relating to receiving and delivering integrated online CBT. Although quotes from supervisors are not included here, their data were analysed and are presented alongside the therapists' accounts, as their views were similar.

### 3.1 Initiating the Therapeutic Alliance: Assessment Video Call Session

Both patients and therapists felt the initial videocall session was crucial to establishing the therapeutic relationship. Therapists also found this mode effective for conducting the preliminary assessment and seeing how the client ‘presented’:

They have most of the benefits of seeing someone in‐person, because … you can hear them, you can see … their environments … You can see how they're dressed, their appearance, and how they look when they're talking to you. A lot of really helpful information.

(Therapist 02)

Patients explained how meeting their therapist face to face meant that they were ‘not a stranger’, and this supported the subsequent sessions mediated by instant messaging:

It was quite helpful to have a face that I could then associate my therapy with later, and it didn't feel quite so [pause] distant. I think perhaps that was why the conversations later on the instant messaging worked, because I knew who I was typing to.

(Female patient, 23 years, 9 sessions)

A minority of patients chose to keep their camera off during the initial videocall session. Therapists assumed this was either due to anxiety, avoidance, technical issues or broadband constraints. However, patients who had kept their camera off explained they struggled speaking about their mental health and did not want to be seen crying.

Some therapists and patients felt more videocalls would have been helpful. They suggested either switching to videocall if a typed session was especially difficult or having the option to use this mode halfway through and/or for the final therapy session. Therapists explained how they prepared their patients during the videocall session by setting expectations about the content and tone of typed messages:

I realize that when people are just getting my typing back their mood is going to influence how they read my words. So, something that was useful from having an initial camera (videocall) was … I'd say to them, ‘I'd like you to try and remember how I'm speaking now and if I challenge you, I'm not doing it in a critical or angry manner’. I think that's the danger with typed therapy. You need to be sure that people aren't reading our words with their mood, putting their mood onto it.

(Therapist 01)

### 3.2 Communication via Typing: Impact on CBT and the Therapeutic Relationship

Some patients adjusted more easily than others to therapy sessions mediated by typed instant messaging, and no age‐related differences were noted. Most reported that typing got easier over time, as they became more familiar with expressing themselves this way. However, a minority struggled with technical issues or to express themselves via typing, especially a patient with dyslexia:

I personally find it difficult translating from my head to paper … because of my dyslexia it's so hard to try and say what I want in a few words … and then it's kind of like [sighs] I've got to make sure that I've got the punctuation right or am I using the right words, does this explain what I'm on about?.

(Female patient, 25 years, 8 sessions)

Therapists with no previous experience of delivering CBT via instant messaging felt they were ‘learning on the job’ (Therapist 02). In typed messages, some therapists purposefully modelled making mistakes to mitigate patients' anxiety about spelling and grammar. Therapists learnt to ask more focused questions, which they felt encouraged them to be more considered about what would be most useful in a session:

So, doing it in the instant, typed platform really forces you to strip it back to the core essence of what I'm trying to say or what I'm trying to ask … which was good! I think it helped me grow a bit as a therapist and be a bit more slowed down and a bit more considered … and really focus on what is the most useful line of questioning or the most useful thing to do in that session.

(Therapist 06)

The pace of typed messaging impacted on the session agenda. Therapists reported reducing their patients' expectations about what could be covered in the sessions compared to in‐person CBT. They felt that they were able to offer patients a ‘positive experience of therapy’ (Therapist 06) with the aim for one significant change in their depressive symptoms. Once patients were accustomed to the pace of typed sessions, some focused in advance on what they wanted to discuss:

The typing got easier and I think for me it was a matter of sort of thinking beforehand and planning well what do I want to get out of this session now. Whereas perhaps if I'd gone straight into a face‐to‐face I might not have thought so deeply about it.

(Male patient, 63 years, 11 sessions)

Both patients and therapists reported frustrations with typed communication. A flashing message indicated when the other person was typing, although this did not always show up on smartphones. Periods of silence led to uncertainty on both sides, as to whether the other person was thinking or distracted. Patients frustrated by delays sometimes became preoccupied by other tasks. Therapists were concerned about patients being distracted, especially if they joined sessions whilst doing other tasks, such as work or childcare. Some patients found typing ‘impersonal’ and a few felt at times they were speaking to a robot or ‘any therapist’ (male patient, 47 years, 2 sessions).

For therapists, one of the biggest challenges was the lack of non‐verbal cues, making it harder to monitor patient distress. They developed strategies to mitigate this, such as regularly asking about patients about their emotional state. Some patients found it hard to express themselves and to convey emotional tone, stating that typing felt ‘more cognitive than emotional’ (female patient, 49 years, 7 sessions). Other patients found that typing allowed more time to think and consequently they experienced less pressure than when speaking. For some patients, the sense of anonymity was conducive to being more open than they would in an in‐person session:

Some people tell you a lot more—and I had a client say that—‘I can expose them (thoughts) and be more vulnerable because I'm typing, and I can't see you’.

(Therapist 04)

Therapists also reflected that typing really suited some people, such as those who were more introverted, or less comfortable with face‐to‐face communication, which was corroborated by this patient:

If you're a very emotional and empathetic person ‐ you need to see people, and you need to read their face a lot. I think it's super good for introverted people who really struggle with human interaction, because typing is their preferred method, they control the environment, they type their articulated message instead of speaking, which for them is difficult, and the structure can be controlled entirely.

(Male patient, 27 years, 9 sessions)

Therapists valued the ability for both themselves and their patients to scroll back and review what had been discussed previously:

It became easier to guide the client to make the most of the typed treatment, because one of the really good things is that you can ask them to scroll up and read back over what they said, if they say something contradictory, or just learnt something new.

(Therapist 02)

### 3.3 Using Platform Resources in Sessions

During early therapy sessions, some therapists found it challenging to provide psychoeducation using typed instant messaging but reported that sharing the platform resources, such as videos and information sheets, supported this aspect of CBT. Also, a few felt that instant messaging was conducive to introducing the educational aspects of CBT:

I think typed therapy can work because it's (CBT) often about skills, learning and educational kind of therapy, so I think that's why this format works quite well, those are the positives. It lends well to, a therapy that is about introducing psycho‐education, skills, interventions that can help somebody that they can go and practice and implement and embed.

(Therapist 03)

Both patients and therapists appreciated having resources stored in one place, which were immediately accessible and provided structure to therapy sessions:

When she was sharing the worksheets it's there instantly and you can … have a quick look and it's also there to look at a bit later on. Instead of having pieces of paper handed to you which you probably end up losing, it's all in one place.

(Female, 62 years, 10 sessions)

Sometimes connectivity issues interfered with completing worksheets. Patients found the small screen on mobile phones made it hard to concurrently fill in worksheets and maintain the chat function. Therapists were frustrated that they could not write in worksheets during the session but used strategies to complete worksheet collaboratively (e.g. used the chat function to agree the content of a box). However, therapists highlighted the patient retained more ‘ownership’ of the worksheet when it was written in their own words:

I found it a bit frustrating that the therapist wasn't able to put things onto the worksheets … but I think it is really useful because they were filling out the worksheet. It was their words whereas sometimes as a therapist you are filling out, say a cross section formulation for example, you are interpreting what they are saying and then putting that in there, whereas if they are filling out their own cross section formulation it can be really helpful that they have got ownership of that and they are putting it in their own words.

(Therapist 07)

Therapists found many patients presented with both depression and anxiety, which was a challenge because most of the platform resources were geared towards treating depression. Some therapists described strategies to address this, such as using thought records to address anxious, rather than depressive thoughts. Some therapists highlighted that core depression focussed CBT activities, such as behavioural activation or behavioural experiments were more challenging via typing. Despite this, some therapists said that having depression‐specific resources helped them stick to Beckian CBT:

Actually, it helps you to stick to the model more when you have got the worksheets. That is one of the things I did find on the study as a therapist that actually I was able to stick the model way more … because that was the main focus, the Beckian CBT … it made it easier to not have that drift as much.

(Therapist 07)

### 3.4 Between Session Tasks and Using the Platform Between Sessions

Therapists reflected that the variation in patients completing ‘between session’ or homework tasks was similar when delivering CBT in person. Patients who did not complete homework explained they found the tasks overwhelming or an unwanted reminder that they were depressed and receiving therapy. Not completing tasks made patients feel anxious about letting the therapist down or wasting their time, and some said this made them want to avoid the next session. Other patients found the platform conducive to homework, as they were able to look back through session transcripts, to re‐read the therapist's explanation of how to complete the worksheet. Also, some patients reported using their smartphone for homework tasks to allow more immediate capturing of thoughts. However, some patients completed homework tasks offline, either on paper or in a written journal, which they found more accessible, as they did not have to log into the platform:

I logged in for the diary, I set up the diary and did some of the diary work in there … But then … because I'd been doing my note‐booking (offline) I was making notes in there as well. So there came a point where I didn't need to go online to do it, I was just using it on my hard copy and that was easier to refer to then.

(Male patient, 63 years, 11 sessions)

It was useful that therapists could check and comment on worksheets when patients completed them on the platform. They suggested that patients who consistently used the platform in between sessions were likely to benefit more from therapy:

For the people that did really well I got loads and loads of worksheets back, two or three a week and for people that didn't engage as well, you could see they'd logged on just before the session and sent one back. So, there wasn't much information there. So, again it's down to motivation I think and people really wanting to acquire the skills and practice out of session, so it's sort of a motivational thing.

(Therapist 09)

Both patients and therapists appreciated being able to re‐read and reflect on transcripts between sessions. This allowed therapists to recall specific things about the patient during supervision and identify how to focus their treatment. When in a different mindset, patients were able to look back and review transcripts, notice their changing thought patterns and recall the therapist's advice:

I've done that on a few occasions where I've thought back and, oh, what did I say? What did my therapist say? It's really helpful to be able to go back. Or even just to be able to compare a different mindset than I have … that transcript is really helpful to be able to revisit that and think, oh, well, that's the frame of mind I was in the time, etc, it's helpful when you're trying to spot a pattern.

(Female patient, 31 years, 11 sessions)

One patient felt that typed sessions were very slow but after reviewing session transcripts was ‘amazed how much ground we had covered’ (male patient, 63 years, 11 sessions). Not all patients used this, and some did not realise they were able to log back in after their course of therapy had ended.

### 3.5 Reasons for Disengaging With Therapy

Therapists did not always know why a patient disengaged from therapy, even when they sensed they were losing them:

It can feel like you're losing someone and you're not quite sure why, whether it's something to do with the relationship, whether it's because they're just not enjoying the platform and this style of therapy.

(Therapist 04)

Patients who had disengaged explained their decision had related to personal circumstances or life events or, more often, because they experienced the CBT approach as too structured or mentally draining. For some, completing worksheets and homework tasks felt too onerous and like extra work, which in turn had a negative effect on their mental health:

I was finding it more draining than anything else. I found it quite mentally hard … I explained how I was feeling, and how it was a lot more work than I was expecting, ‘It's making me feel quite bad, and I just don't need this extra level of work and responsibility’.

(Female patient, 27 years, 3 sessions)

Patients also disengaged if they disliked typing because they found it hard to express thoughts or feelings or were anxious about spelling and grammar. Some cited confusion about overlapping messages, frustration when waiting for replies and the impersonal sense that their therapist could be anyone. One therapist wondered whether patients disengaged because they could not see their therapist and consequently felt less personal connection to them:

I just think it's something about the way it felt a bit more casual … by not going into a particular space, by not having that human contact. I sometimes felt like I wondered if they didn't think I was a person, because they didn't see me a lot of the time and I sometimes wondered if it was easier for them to not attend because I wondered if they didn't feel like they were letting me down, they were letting down a robot!.

(Therapist 04)

### 3.6 Reasons for Completing Therapy

Patients who completed therapy generally appreciated the combination of talking to a therapist and accessing resources within one platform. They reported learning about the patterns of depression and specifically how to manage negative thoughts:

I suppose it's the combination of all … I think it was being able to read what she was talking about, and what she was thinking … because she would then say, ‘Well, this is something that might be useful’, for me to read, and then I'd read it and if it was helpful, which most of the time it was, I would then have a better understanding of what I was experiencing. Being able to work on that in my own time, worked quite well with how I think. I think as a combination it worked effectively for me.

(Female patient, 22 years, 9 sessions)

Some were still using ‘tools’ which they had learnt during therapy and valued the ability to log back into the platform after finishing therapy, allowing patients to see ‘how far they had come’ (female patient, 32, 11 sessions) and to consolidate their learning. Some felt that the therapeutic relationship was the key element in guiding and supporting changes in their depression management:

The important thing was having the therapist that you were accountable to, to go back to it and to be accountable for thinking about it, talking about it … it's not just reading it, but it's sort of putting it into action. Something you can put into action, much of this stuff can be in the head but then whether you actually go and do it with a proper plan is another thing.

(Male patient, 63 years, 11 sessions)

## 4 Discussion

### 4.1 Summary of Findings

Delivering CBT that combines therapist led sessions and CBT resources within one online platform is acceptable and beneficial for some people with depression. Many patients learnt skills to manage their depression and attributed this to the relationship with their therapist and the skills they acquired through using the online resources. Therapists believed that therapy delivered via the platform was a good first step for people to learn CBT skills. They recognised that the depression‐focussed resources helped to structure sessions but could be challenging when patients wanted support for other co‐morbidities, such as anxiety. The findings show that specific attributes of the platform which supported therapy were the ability to scroll back through transcripts during sessions and to review these between sessions. Completing homework tasks on the platform was also valuable and enabled the therapist to monitor engagement, but not all patients used the platform between sessions.

The findings suggest that therapy mediated by instant typed messaging is a suitable option for patients who are willing and able to express themselves via typing and who are comfortable (or prefer) communicating with little or no face‐to‐face contact. Patients who liked the typed format experienced less pressure than in a face‐to‐face setting (in‐person or via videocall), which allowed more time to think and respond. For other patients, the slow speed of communication and difficulty expressing themselves in written words was frustrating. The typed format led therapists to hone their skills by using focussed questions, shape their patients' expectations about the most effective use of session time and employ strategies to mitigate the lack of nonverbal cues and monitor for distress.

The therapeutic relationship was established effectively via an initial videocall assessment. Whilst most patients and therapists went on to develop and maintain an effective therapeutic relationship, the issues associated with typing sometimes caused difficulties. Patients who did not complete therapy disliked the CBT approach, finding the worksheets and between session tasks onerous. Many also found therapy mediated via instant typed messaging to be challenging and sometimes anxiety provoking. Some avoided the process as a result. Some found it hard to express themselves, were confused by overlapping messages and frustrated with the time taken waiting for replies.

### 4.2 Comparison to Other Literature

Our study supports previous work showing that online CBT with therapist guidance and online therapy mediated via instant typed messaging is acceptable for some patients with depression [6, 7, 8]. These patients are likely to be comfortable expressing themselves via typing, value the opportunity to review and reflect on written transcripts and motivated to engage in the tasks required by the CBT approach [6]. Previous work identifies key design features for online CBT, highlighting the importance of face‐to‐face sessions to build rapport and trust [18]. We ensured a face‐to‐face meeting via the initial videocall, and this was found to be important for both patients and therapists in establishing the therapeutic relationship.

Access to CBT worksheet resources is an important mediator of outcome [13, 14], and we found this function was appreciated both during and in‐between sessions. Our findings suggest that during sessions time could be used more effectively if both parties could collaboratively complete worksheets via a shared online tool. In between sessions, access to CBT resources is crucial to support homework tasks [12, 13, 14], the completion of which is important to facilitate treatment outcome [25]. Our findings show that patients valued being able to review transcripts as a reminder of how to complete their homework tasks and provide examples of some patients using their smartphones for homework tasks (e.g., the notes function), allowing them to record immediate thoughts and experiences. However, logging into the platform between sessions required motivation, and some patients chose to complete homework tasks offline. Previous research suggested that engagement with homework tasks could be improved by ensuring CBT resources can be accessed on a smartphone [14].

Our study supports research that highlights the importance of establishing patient preferences regarding the mode of CBT delivery and ensuring that referrals are targeted accordingly [6]. Brief videos could inform patients about what options exist and empower them to make informed choices. This might affect engagement and drop‐out. It also extends this by emphasising the importance of establishing patient and therapist expectations about what can be achieved in sessions mediated by typing.

## 5 Strengths and Limitations

The patient sample included an even mix of male and female participants, who varied in terms of age, depression severity and a balance of those who did and did not engage well with this mode of CBT therapy. However, all participants (patients, therapists and supervisors) had agreed to be involved in a trial of integrated CBT and were therefore open to the idea of delivering or receiving CBT via an online platform. Therefore, their views might not be representative of their peers.

The intervention was being assessed within a trial context, and therefore, therapists had less flexibility in how it could be used and were limited in the CBT resources they could share. If the platform resources could have been used in a more flexible way, which would probably be the case in real‐world setting, some of their concerns might have been addressed.

## 6 Conclusions

Integrated online CBT is acceptable to some primary care patients with depression. Both patients and therapists felt less could be covered in a therapy session mediated by instant messaging than in‐person but that the slower pace allowed reflection and a more focused discussion. While some patients struggled with instant messaging and did not access the integrated online resources, others benefited from receiving support from a therapist whilst also having access to resources which, together, enabled them to learn skills to manage their depression. There were additional benefits, as the platform allowed patients to read and review transcripts to recall what had been said, better understand how to complete the homework and track how they had progressed. A conversation that sets out realistic expectations about therapy sessions mediated by instant messaging and the benefits and challenges of this integrated approach will be important in ensuring that patients are able to make an informed choice about this novel mode of delivery of CBT. This will be key to maximising engagement.

## Author Contributions

**Fiona Fox:** methodology, writing–review and editing, formal analysis, data curation, writing–original draft. **Nicola Wiles:** conceptualisation, investigation, funding acquisition, writing–review and editing. **David Kessler:** conceptualisation, investigation, funding acquisition, writing–review and editing. **Debbie Tallon:** investigation, funding acquisition, writing–review and editing, formal analysis, data curation, project administration. **Laura Thomas:** writing–review and editing, project administration. **Christopher Williams:** conceptualisation, writing–review and editing, funding acquisition, resources. **Roz Shafran:** funding acquisition, conceptualisation, writing–review and editing. **Paul Lanham:** conceptualisation, writing–review and editing. **Katrina Turner:** conceptualisation, methodology, funding acquisition, supervision, writing–review and editing, formal analysis.

## Ethics Statement

As part of the approvals for the main trial, ethical approval for the qualitative study was granted by the Southwest—Frenchay Research Ethics Committee (REC reference: 19/SW/0038) and Health Research Authority (HRA).

## Conflicts of Interest

C.W. is Director and shareholder of Five Areas Ltd, which licensed worksheet components for use in the INTERACT integrated CBT package, and is also author of written and online CBT self‐help resources addressing depression and anxiety. The other authors have no conflict of interests with respect to this paper.

## Supporting information

Supporting information.

## Data Availability

Data from the INTERACT trial will be lodged with the University of Bristol Research Data Repository and assigned a unique DOI. Access to INTERACT data for non‐commercial research will be through a system of managed access (University of Bristol ‘controlled access’). The final decision to release data will be taken by the University of Bristol's Data Access Committee. There may be a charge for preparing the data set. Requests for data any other purposes must be negotiated separately.
