# Supplementary material for: Patients' and Therapists' Views of Integrated Online CBT for Depression
Source: Health Expect. 2024 Aug 21;27(4):e70002. doi: 10.1111/hex.70002 (PMC11339129; doi:10.1111/hex.70002)
Supplement: Supplementary file 1 — Supporting information. [file HEX-27-e70002-s001.docx]

# **Interview Topic Guide 1: Intervention group**

## Current situation and health status

- Just to give me some context, please can you briefly tell me a bit about yourself, for example, who you live with, what you do?

**Previous mental health** (Depression history / Treatment pre-trial)

- Can you tell me a bit more about what your depression when did it start, how did it affect you?
- Before this trial how did you have any talking therapy / CBT specifically?
- Can you recall how things were around the time that you were invited to take part in the study?

**Motivation to take part in study and experience of randomisation**

## Experience of integrated CBT

- What do you think about CBT being delivered to patients using an integrated approach, i.e. online materials with input from a therapist?
  - Prompt for what they saw as the benefits and disadvantages; what they found helpful/unhelpful; what impact they think it had on their depression, how effective they think it is as a treatment (in itself and compared to face to face CBT) and as a way of therapists and patients working together
- How did you find working with a therapist through the platform?
  - Prompt for how they think working online (rather than in person) affected the client-therapist relationship in terms of how they communicated, interacted, and what areas were discussed How did you communicate with your therapist, e.g. IM online, by phone, face-to-face in person, by videocall?
- How did these different ways of communication affect your relationship with the therapist?
  - Prompt: what was your experience of using the chat; what was your experience of using video and phone; what influenced what mode was used; what did you use each mode for; did you switch between different modes in a single session; what did you like and dislike about each mode of communication; how did they differ in terms of what you discussed, how you processed and presented your thoughts; ease of use; ability to reflect on what was being discussed and process information; take in what the therapist was saying and suggesting?
- Platform – how easy or difficult to navigate?
- What did you think of the worksheets, information sheets and videos? Which did you use, when and why?
- How did you use them?
  - Prompt about their experiences of working with the therapist and working on their own between sessions and how this was done: did they work on worksheets with the therapist in sessions; if so, how did they do this and did they start/share and discuss worksheets in sessions; did they work on them independently; who decided which worksheets would be used and when?
- What did you find helpful/unhelpful about the worksheets, information sheets and videos? How well did each of these materials support working with the therapist and working independently between sessions?
- What elements of integrated CBT treatment did you think benefited your mental health most and why?
- Talking to a therapist, completing tasks, reading information sheets etc. How did each affect you and enable you to understand your depression and learn ways to manage it?

How did you view yourself/your role in the therapy? Did you view yourself as working with a therapist and/or learning from a therapist? Agenda setting

- What new skills/knowledge/practices do you think you now have because of INTERACT? Do you now use them in your daily life?
- What did you think about the balance between the number of face-to-face (in person/videocall) and online sessions?
- What did you think about the frequency and length of the online sessions?
- How many online sessions did you have?
  - If less than the total number allowed, asked why they stopped treatment early. If they had all the sessions, ask if they felt they had needed more.
  - Starting and ending therapy what was your experience?
  - Logging back in – reviewing notes / transcripts?
  - Endings of sessions how was this managed?
  - Convenience and flexibility – sessions in public or private? impact on quality?

## Other treatment in the trial and current treatment

- What care did your GP give you? How often have you seen him/her in the last 6 months? Is this more, less or the same as before you started taking part in the study? Prompt for ADs, medication, Impact of medication; referrals to talking therapies
- During your time in the study, what other treatment/support did you receive?
  - cCBT; other counselling, CBT, support from others (friends, family, GP)
  - Were they helpful or not?
- How do your current symptoms of depression affect you compared to when you started the INTERACT study? Do you think that your depression has improved since starting the study? In what ways?
- Apart from treatment received during the study, what other things have affected your mental health during your time in the study?
- Prompt for both positive and negative factors

## Views of the study

- What was your experience of the calls and follow ups throughout the study?
- What do you see as the advantages and disadvantages to taking part in the INTERACT study?
- Are there any other comments you would like to make about the study or treatment received?

**Interview Topic Guide 2: Therapists**

**BACKGROUND**

- Can you tell me a bit about yourself? Your clinical background and work experience; what types of therapy you usually deliver, what type of clients you usually see? Have you supervised other therapists?
- Before INTERACT, had you used online platforms or provided support alongside computerised or online CBT treatments? If yes, what was your experience of delivering therapy this way?

**VIEWS ABOUT INTEGRATED APPROACH**

- What do you think about delivering CBT to clients using an integrated approach, i.e. online materials with input from a therapist?
  - Prompt for what they think about collaborative online therapy and working on materials together, what they see as the benefits and disadvantages for therapists and clients; what they found helpful/unhelpful; how effective they think it is as a treatment (in itself and compared to in-person CBT) and as a way of therapists and clients working together
- What impact do you think it has on how CBT is delivered?
- What would you say are the key strengths and weakness of using an integrated approach?

**DELIVERING INTEGRATED ONLINE CBT**

1. **COMMUNICATION MODES**

- How did you find working with clients remotely?
- What did you think about having the first session via videocall? Did clients have cameras on? If not why not and how did it affect the session?
- What was your experience of the initial videocalls?
- What was it like then switching to communicating in sessions via typing?
- What was your experience of using typing? Did you find this easy / difficult? Did this change from your first few clients to later clients? If so, how?
- What impact do you think it had on what points you raised, how you spoke to and communicated with clients?
- How much you were able to cover in a session compared to a “standard” in-person individual therapy?
- Were there any issues with your / your client’s typing speed or delays in replying?
- Did you use any strategies to improve the time / delay? Probe - did you have stock responses eg. copy and paste? If so when did you use them? How useful / effective?
- How did typing affect the content of therapy or type of homework?
- How did it affect client’s engagement?
- Did you ever switch to another communication mode? Eg. videocall or phone? If so, why and when?
- What did you think about the balance between the number of videocalls and typed sessions?
- What did you think of the total number of sessions allowed, their frequency and length?
- Did you communicate with clients between sessions? If so, how did you do this. eg. re-arranging missed sessions. And if didn't do this via platform messaging system - why not? What were the limitations?

**PLATFORM RESOURCES:** Worksheets, information sheets and videos

- How did you use Information sheets? Used during therapy sessions / between sessions?
- How did they compare to information sheets you normally use?
- How often did you use videos? Used during therapy sessions / between sessions?
- How helpful/unhelpful did you find them? How did your clients engage with them?
- How did you use the worksheets with your clients?
- Did you introduce them during a session with a client or share with clients to complete as part of the between-session tasks? When did you give feedback/comment on the worksheets?
- Is there anything you think could be improved in terms of functionality and how they work/are shared/can be edited?
- Did you use more or less worksheets than you would normally use when providing standard in-person individual CBT?
- Was it easier or harder to use the integrated resources compared to using paper worksheets?
- How did the resources work together, as a group of resources?
- How did using these materials fit in with your previous way of working?
- In what ways (if any) did you adapt or customise the way you used the *INTERACT* therapy platform compared with how it was introduced in the initial training sessions?

**RELATIONSHIP: THERAPIST / CLIENT**

- Did the integrated approach affect your relationships with clients? If so how?
- Did delivering CBT online impact on issues such as trust? If so how?
  - How did you recall specific issues/details about individual clients without seeing them?
  - How did your relationships with clients compare to in person CBT?
  - Thinking about the modes of communication you used, how do you think they affected your relationship with the clients? How did they affect their engagement?
  - How did you manage clients who frequently missed sessions or cancelled at short notice?
  - How did the trial's DNA policy inform your approach? Was it helpful?
  - How did it align with your approach to managing such issues with clients previously (e.g. NHS/private practice)

**GENERAL ISSUES**

- Is there anything about the approach as a whole or the platform specifically that you would change?
- Do you think the training you received was sufficient and prepared you to deliver integrated CBT?
- What did you think about the supervision you received, was it sufficient and appropriate?
- What was your experience of working as a therapist on a research study?
- Do you have any comments or thoughts about the INTERACT study as a whole?
- Do you have any final comments or observations?

**Interview Topic Guide 3: Supervisors**

**Background**

- Can you tell me a bit about yourself?
- Your clinical background and work experience
- what types of therapy you usually deliver
- what type of clients you usually see
- have you previously supervised therapists?
- Before INTERACT, what was your knowledge and experience of online platforms or cCBT?

**Integrated approach**

- What do you think about delivering CBT to clients using an integrated approach, i.e. online materials with input from a therapist?
  - Prompt to discuss different types of online materials (e.g. information, worksheets) and different modes of communication (e.g. face-to-face/video, in-session chat).
- What impact do you think it has on how therapists deliver CBT?
- How do you think it affects the relationship between therapists and clients?
- What do you think about the number, duration and frequency of sessions?

**Supervision of therapists**

- How did you deliver supervision to the therapists during the study?
- How did the integrated approach affect what was discussed in supervision and how you supervised therapists?
- How do you think the therapists used the platform? Where there any differences in their approach you noticed?
- Based on what the therapists told you, what additional issues do you think using an integrated approach and platform raised for them? How did it support/hinder the work they did with clients and with you?
- What types of information did you access when supervising the therapists, e.g. did you look at worksheets that clients had completed, viewed PHQ-9 scores, etc.
- Did you use the platform?
  - If yes:
    - How did you use it? E.g. between or during supervision sessions?
    - How useful was it to have access to the platform to facilitate supervision sessions?
    - Did you encounter any difficulties with the platform?
  - If no:
    - Could you explain why you didn’t use the platform to support supervision sessions?
- Do you think the frequency and duration of the supervision sessions was adequate/inadequate?

**General comments**

- In its current form, what would you say are the key strengths and weakness of using an integrated approach?
- Is there anything about the approach as a whole or the platform specifically that you would change?
- What are your views about an integrated platform like this being rolled out into the NHS?
- And finally, what did you think of the INTERACT study as a whole? How was your experience of being part of this study?
- Do you have any final comments or observations?
